# Publisher Correction: Tracking cryptic SARS-CoV-2 lineages detected in NYC wastewater

**DOI:** 10.1038/s41467-022-29573-1

**Published:** 2022-03-30

**Authors:** Davida S. Smyth, Monica Trujillo, Devon A. Gregory, Kristen Cheung, Anna Gao, Maddie Graham, Yue Guan, Caitlyn Guldenpfennig, Irene Hoxie, Sherin Kannoly, Nanami Kubota, Terri D. Lyddon, Michelle Markman, Clayton Rushford, Kaung Myat San, Geena Sompanya, Fabrizio Spagnolo, Reinier Suarez, Emma Teixeiro, Mark Daniels, Marc C. Johnson, John J. Dennehy

**Affiliations:** 1 Department of Life Sciences, Texas A&M University-San Antonio, San Antonio, TX 78224 USA; 2 Department of Biological Sciences and Geology, Queensborough Community College of The City University of New York, Queens, NY 11364 USA; 3 Department of Molecular Microbiology and Immunology, University of Missouri-School of Medicine, Columbia, MO 65212 USA; 4 Biology Department, Queens College and The Graduate Center of The City University of New York, Queens, NY 11367 USA; 5 Department of Biological & Environmental Sciences, Long Island University–Post, Greenvale, New York, 11548 USA

**Keywords:** Water microbiology, SARS-CoV-2

Correction to: *Nature Communications* 10.1038/s41467-022-28246-3, published online 03 February 2022.

The original version of this Article contained an error in the Data Availability, in which the accession code for ‘NCBI’s Sequence Read Archive (SRA)’ was omitted. The correct version adds ‘PRJNA715712’ after ‘accession #’. This has been corrected in both the PDF and HTML versions of the Article.

