# Supplementary material for: Tracking cryptic SARS-CoV-2 lineages detected in NYC wastewater
Source: Nat Commun. 2022 Feb 3;13:635. doi: 10.1038/s41467-022-28246-3 (PMC8813986; doi:10.1038/s41467-022-28246-3)
Supplement: Supplementary file 1 — Supplementary information [file 41467_2022_28246_MOESM1_ESM.docx]

**Supplementary Information for “*Tracking Cryptic SARS-CoV-2 Lineages Detected in NYC Wastewater”***

**Contents**

Pg. 2 – Supplementary Table 1. Primers and probes used in this study.

Pg. 3 – Supplementary Table 2. Viral N1 copies/L by date for WWTPs associated with unknown lineages described in Figure 1C.

Pg. 4 – Supplementary Figure 1. Viral titer (SARS-CoV-2 N1 copies per L) at each sampling date is plotted against NYC confirmed clinical cases (7-day average) over the sampling period January 1 to June 28, 2021.

**Supplementary Table 1. Primers and probes used in this study.**

| **Name and Site** | **Forward Primer (Probe)** | **Reverse Primer** | **Source** |
| --- | --- | --- | --- |
| 2019-nCoV_N1 (SARS-CoV-2 spike) | GACCCCAAAATCAGCGAAAT | TCTGGTTACTGCCAGTTGAATCTG | CDC 2019-nCoV Real-Time RT-PCR Diagnostic Panel |
| 2019-nCoV_N1 Probe (SARS-CoV-2 spike) | FAM-ACCCCG CAT/ZEN/TACGTTTGGTGGACC-3IABkFQ |  | CDC 2019-nCoV Real-Time RT-PCR Diagnostic Panel |
| iSeq 100 RBD sequencing primers  (SARS-CoV-2 spike receptor binding domain)* | tcgtcggcagcgtcagatgtgtataagagacagCCAGATGATTTTACAGGCTGCG | gtctcgtgggctcggagatgtgtataagagacagGAAAGTACTACTACTCTGTATGGTTGG | This study |
| MiSeq RBD primary PCR primers  (SARS-CoV-2 spike receptor binding domain) | CTGCTTTACTAATGTCTATGCAGATTC | TCCTGATAAAGAACAGCAACCT | Reference 8 |
| MiSeq RBD Nested PCR primers  (SARS-CoV-2 spike receptor binding domain)* | acactctttccctacacgacgctcttccgatctGTGATGAAGTCAGACAAATCGC | gtgactggagttcagacgtgtgctcttccgatctATGTCAAGAATCTCAAGTGTCTG | Reference 8 |
| 12S-V5-Tailed-F1 and R1 | TCGTCGGCAGCGTCAGATGTGTATAAGAGACAGACTGGGATTAGATACCCC | GTCTCGTGGGCTCGGAGATGTGTATAAGAGACAGAGAACAGGCTCCTCTAG | Reference 43 |
| MiSeq 12s PCR primers* | acactctttccctacacgacgctcttccgatctACTGGGATTAGATACCCC | gtgactggagttcagacgtgtgctcttccgatctTAGAACAGGCTCCTCTAG | Reference 43 |

***adaptor sequences are indicated in lowercase letters**

**Supplementary Table 2.** Viral N1 copies/L by date for WWTPs associated with unknown lineages described in Figure 1C.

| **Date** | **WWTP 3** | **WWTP 10** | **WWTP 11** |
| --- | --- | --- | --- |
| 01/31/21 | 1,496,155.0 | N/A | N/A |
| 02/28/21 | 464,927.1 | 40,046.3 | 240,540.3 |
| 03/14/21 | 302,431.6 | 81,635.5 | 158,205.5 |
| 04/05/21 | 149,385.9 | 121,498.9 | 280,317.7 |
| 04/19/21 | 61,092.8 | 72,645.4 | 49,146.5 |
| 05/10/21 | 8,183.5 | 8,085.3 | 117,408.2 |
| 05/26/21 | 12,978.3 | 28,591.6 | 130,956.9 |
| 06/07/21 | 8,812.9 | 18,082.1 | 19,436.2 |
| 06/14/21 | 17,366.4 | 165,067.6 | 4,273.3 |
| 06/28/21 | 12,639.4 | 47,753.7 | 101,757.1 |

**Supplementary Figure 1.** Viral titer (SARS-CoV-2 N1 copies per L) at each sampling date is plotted against NYC confirmed clinical cases (7-day average) over the sampling period January 1 to June 28, 2021.

**
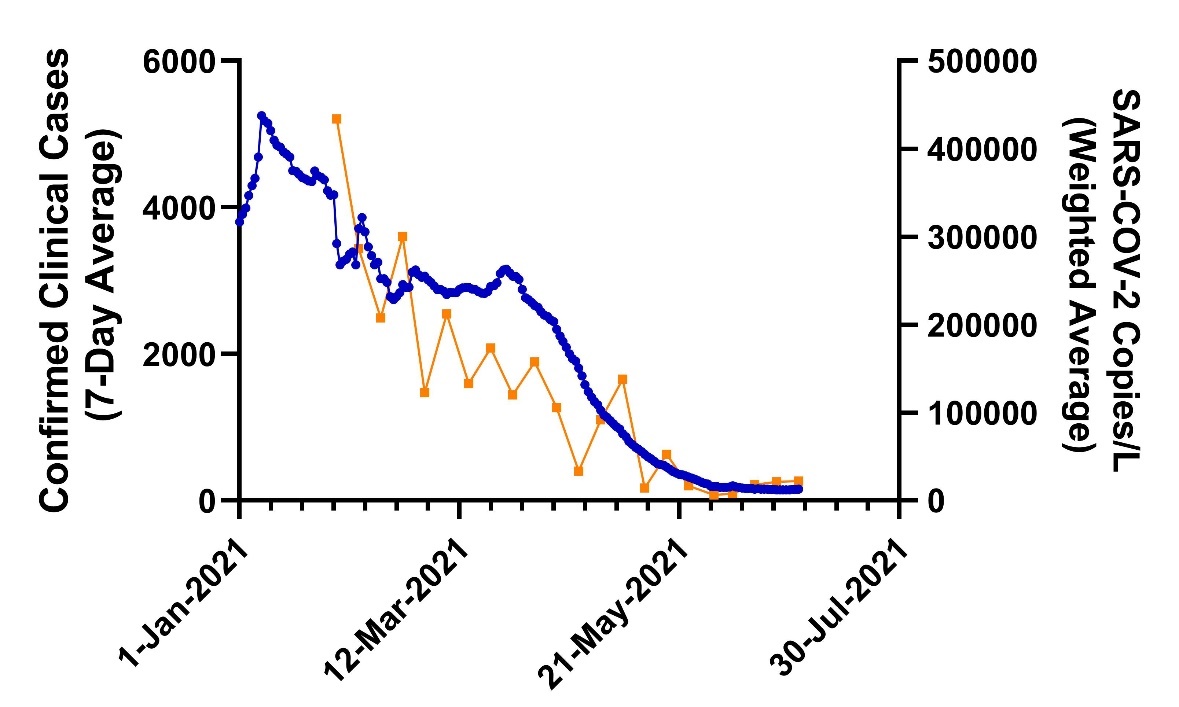
**
