# Supplementary material for: Tracking cryptic SARS-CoV-2 lineages detected in NYC wastewater
Source: Nat Commun. 2022 Feb 3;13:635. doi: 10.1038/s41467-022-28246-3 (PMC8813986; doi:10.1038/s41467-022-28246-3)
Supplement: Supplementary file 3 — Description of Additional Supplementary Files [file 41467_2022_28246_MOESM3_ESM.docx]

**Description of Additional Supplementary Information**

Title: Supplementary Data 1

Description: Mutation frequencies with respect to Wuhan reference sequence (NC_045512.2) in iSeq sequenced samples assayed between 2021/01/31 and 2021/03/14.
